# Impact of farmer professional cooperative on safety production behavior in terms of quality and safety of agricultural products

**DOI:** 10.3389/fpubh.2022.914867

**Published:** 2022-10-19

**Authors:** Yun Teng, Xinlin Chen, Mei Zhang

**Affiliations:** ^1^School of Engineering, Northeast Agricultural University, Harbin, China; ^2^Postdoctoral Research Station of Agricultural and Forestry Economic Management, Northeast Agricultural University, Harbin, China; ^3^School of Economics and Management, Northeast Agricultural University, Harbin, China

**Keywords:** quality and safety of agricultural products, standardized farmer professional cooperative, safety production behavior, safety motivation, agriculture green development

## Abstract

“How to realize farmers to actively produce quality and safety agricultural products” has become a common problem faced by researchers and practitioners. Based on the Triadic Reciprocal Determinism theory and Behavior-motivation theory, the study tries to answer this question from the perspective of standardized farmer professional cooperatives in China, and then solve relevant international problems. The empirical results of 767 sample data using SPSS-AMOS methods show that the restraint factors of standardized farmer professional cooperative have positive effects on safety negative behavior and negative impact on safety positive behavior, and the motivation factors of standardized farmer professional cooperatives have positive effects on safety positive behavior. The restraint factors of farmer professional cooperatives have a positive impact on safety controlled motivation and negative impact on safety autonomous motivation, and the motivation factors of farmer professional cooperatives positively affect the safety autonomous motivation. The safety controlled motivation positively affects safety negative behavior and safety autonomous motivation negatively affects safety negative behavior and positively impacts on safety positive behavior. The above findings theoretically make a useful supplement to the study of farmers' safety production behavior, and have guiding significance to the construction of standardized farmer professional cooperatives in the world.

## Introduction

It is urgent to improve the quality and safety of agricultural products. In order to improve the quality and safety of agricultural products, various countries have implemented measures, such as the action plan for harmless food, green organic certification, standards for residue limitation of agricultural products, and standards of residual detection. Although these efforts have greatly improved the safety of agricultural products, the people are also expected “to eat safe agricultural products.” The problem of agricultural product safety has become increasingly intense. The results of the “alternative project” of highly toxic pesticides showed that more than 50 thousand people were poisoned and killed every year because of the irrational use of drugs and the use of highly toxic pesticides, such as cabbage, ginger, poisonous leek, and cowpea, which seriously affected and threatened the health of the people and social stability, and aroused great concern in all walks of life. In particular, it emphasizes that the safety of food should be managed from the source, from the producers of agricultural products to ensure the people's safety.

Promoting farmers to actively produce the quality and safety of agricultural products is the key to improving the quality and safety level of agricultural products. The quality and safety of goods should be controlled at the source and by the producers of agricultural products. Farmers' production behavior is the direct factor determining the quality and safety of agricultural products (1–10). According to the principle of behavior evolution, some researchers point out that the production behavior of employees under enterprise safety production is an individual behavior with dynamic characteristics, and empirically conclude that under the joint action of many factors, employees change from passive to active in safety production. This study conducts an evolution priniciple extending to the quality and safety of agricultural products under the “farmer production behavior”, based on the actual situation, to study how to promote farmers from passive to active in the production quality and safety of agricutural products. The core idea is that “Farmers are passive to produce quality and safe agricultural products” should rise to “farmers take the initiative to produce quality and safety of agricultural products,” and improve the quality and safety of agricultural products from the source.

The standardized farmer professional cooperative is an effective way to promote farmers' initiative in producing quality and safety of agricultural products. In China, there are three levels of cooperatives: national head office, local cooperatives, and grass-roots cooperatives. The first level is the national head office, which refers to the head office of cooperatives in a country. The second level is local cooperative associations, which refer to cooperative associations established at the local level, such as cooperative associations of provinces (municipalities directly under the central government, autonomous regions) and cooperative associations of counties, etc. The third level is grass-roots cooperatives, which mainly refer to various types of cooperatives established on the basis of resources based on the principle of a cooperative system with producers and operators as the main body in most grass-roots production and operation units. Standardized farmer professional cooperatives mainly include three types: the original cooperatives of “cooperative + farmers,” the company affiliation cooperatives (the “company + cooperative + farmers” organization led by the cooperative), and the corporate-led cooperatives (the “company + cooperative + farmers” organization led by the company). Standardized farmer professional cooperative has three basic characteristics: highly organized unified coordination; oriented to members, motivations, and restraints; providing security and orderly production (11–13). The academic community has gradually formed a consensus that compared with the loose farmer professional cooperatives, the standardized farmer professional cooperatives are more organized and standardized, and their management factors have a stronger impact on Farmers' safe production behavior (14–17). Bandura (18), an authority in the school of social cognition, emphasized in his three-way interaction theory that effective organizational management factors can help break individual negative emotions, transform individual behavior from passive to active, and promote the development of individual behavior to a benign direction. Parker (19), a famous expert on organizational behavior, believes that appropriate organizational management factors can promote individuals to complete their in-role behaviors, which will rise to organizational citizenship behaviors aimed at helping others. Many researchers follow Bandura and Lothers' ideas that organizational management factors influence individual behavior changes. Opportunistic behavior refers to that under the condition of information asymmetry, people do not fully disclose all information truthfully and engage in other behaviors that benefit themselves at the expense of others. Opportunism will bring some risks to farmer professional cooperatives. Farmers do not follow the management of farmer professional cooperatives to carry out green production, which is a kind of egoistic and selfish behavior, and will bring risks to the farmer professional cooperative. Bandura, an authority in the school of social cognition, proposed based on the Triadic Reciprocal Determinism theory that “environmental factors affect individual psychology, and individual psychology affects people's behavior,” and that effective organizational management factors can help break the negative emotions of individuals, transform individual behavior from passive to active, and promote the development of individual behavior to a benign direction. Parker, a famous expert on organizational behavior, believes that appropriate organizational management factors can promote individuals to complete their in-role behaviors, which will rise to organizational citizenship behaviors aimed at helping others. Many researchers follow Bandura and Lothers' ideas that organizational management factors influence individual behavior changes. Through the analysis of the practical situation, it can be seen that the management factors of standardized farmer specialized cooperatives belong to environmental factors, the safety motivation belongs to the psychological factors of farmers, and the safety production behavior belongs to the behaviors taken by farmers. According to the Triadic Reciprocal Determinism theory, it can be preliminarily judged that the management factors of standardized farmer specialized cooperatives affect the safety motivation, and then affect the behavior of farmers. In addition, behavioral motivation theory also better interpretation of this problem, the generation of behavior comes from motivation.

In view of this, this study will focus on the issue of “how to realize the initiative in the production of quality and safety agricultural products by farmers.” According to the Triadic Reciprocal Determinism theory and Behavior-motivation theory, this study will construct a theoretical analysis framework including the antecedent variables of farmer professional cooperatives, intermediary variables of safety motivation, and post-dependent variables of safety production behavior, and conduct empirical analysis. This paper reveals the relationship between standardized farmer professional cooperatives and farmers' production behavior, and provides theoretical reference and practical guidance for promoting farmers' implementation of safe active behavior and scientific management of farmer professional cooperatives.

## Concept definition and research hypothesis

### Concept definition

#### Standardized farmer professional cooperative

Internationally, rural cooperative economic organizations are collectively referred to as agricultural cooperatives. The standardized farmer professional cooperative is a farmer professional cooperative with the function of encouraging and restraining. It is a standardized organization entity that is closely related to the farmers and land. The standardized farmer professional cooperative includes motivation and restraint factors. The motivation factors mainly include providing stable sales channels, training and learning, resistance risk, price guarantee, credit financing, and typical demonstration (20). The restraint factors mainly include strict control of pesticide dosage, unified selection of pesticide and fertilizer types, safety testing, supervision among members, quality standards and punishment for breach of contract, etc. (21–25).

#### Safety production behavior

The connotation definition of safety production behavior. Safety production behavior refers to the activities that affect the quality and safety of agricultural products produced by farmers under various internal and external stimuli in the production process of agricultural products (26, 27). According to the individual initiative level, safety production behavior is divided into safety negative behavior and safety positive behavior. Safety negative behavior refers to harmful behavior that reduces the quality and safety of agricultural products carried out by farmers under the influence of internal and external factors in the process of agricultural production. Safety positive behavior refers to the beneficial behavior that farmers implement to improve the quality and safety of agricultural products under the influence of internal and external factors in the process of agricultural production (28).

#### Safety motivation

The connotation definition of safety motivation. The theory of behavioral motivation holds that human behavior is governed by motivation (29). Motivation is the direct cause of causing and maintaining individual behavior (30). Motivation refers to the idea of carrying out activities to meet certain needs, and is the internal reason that drives people to engage in various activities. The demand level theory proposed by American psychologist Maslow (31) put safety demand into the second level, which means that safety needs are one of the basic needs of people. Under specific circumstances, we should pay attention to the quality of individual motivation rather than the quantity of motivation. Motivation can be divided into autonomous motivation and controlled motivation (32). Autonomous motivation refers to an individual voluntarily or according to their own interests, beliefs, etc., from a certain behavior, controlled motivation refers to an individual due to internal or external engagement in certain behavior. Autonomous motivation and controlled motivation are the reasons for individual behavior, and answer “why do people generate such behavior?” Different types of motivation have different predictive power on outcome variables, and individual behaviors of different motivation types have different outcome effects. Autonomous motivation has a significant positive predictive effect on the individual behavior results, and controlled motivation has a significant negative predictive effect on the individual behavior results. In the process of agricultural production, the farmers' safety motivation can be divided into safety autonomous motivation and safety controlled motivation (6). The former refers to the motivation of farmers to produce quality and safety agricultural products due to their own choice and will, while the latter refers to the motivation of farmers to produce quality and safety agricultural products under external or internal pressure.

### Concept definition and research hypothesis

The Triadic Reciprocal Determinism theory, proposed by the American educator Albert Bandura, refers to the causal interaction among individuals, behaviors, and environments. First, causal interaction between individuals and behaviors. Individuals and behavior interact with each other. Individual perception can stimulate and maintain behavior, and behavior will be affected by individual cognitive level. Due to the difference in individual thinking and cognition, individuals will have different behaviors. The feedback effect of behavior in turn will reconstruct individual thinking and cognition. Second, causal interaction between behavior and environment. Behavior and environment interact with each other. The environment breeds behavior and promotes the production of behavior. Third, causal interaction between environment and individual. Environment and individuals interact with each other.

#### Standardized farmer professional cooperative and safety production behavior

Abhilash and Singh (33) found that the farmer professional cooperative is the main factor affecting the use of pollution-free pesticides, and the more closely related farmers, the more inclined to adopt safety production behavior. The management factors of farmer professional cooperatives mainly include motivation factors and restraint factors. The effective combination of these factors can cause farmers to effectively reduce unsafe production behavior (1). Wang (34) and others, based on the model of agricultural standardization to study agricultural standardization, pointed out that the management factors of standardized farmer professional cooperatives can promote the evolution of the production of agricultural products. Ji et al. (35) and others make an empirical analysis of the relationship between the farmers' safety production behavior and the farmer professional cooperative. Compared with the motivation factors, the restraint factors of the standardized farmer professional cooperative have a greater impact on the farmers' production behavior. Zhao et al. (36) pointed out that the effectiveness of the management factors of the farmer professional cooperative had a significant influence on the standardized pesticide application behavior. Chen (37) confirmed that the regulatory factors of standardized farmer professional cooperatives have significantly promoted the quality control and safety production behavior of the farmers. According to the Triadic Reciprocal Determinism theory, the management factors of standardized farmer specialized cooperatives belong to environmental factors, and the safe production behaviors belong to the behaviors taken by farmers. Behavior and environment interact with each other. The environment breeds behavior and promotes the production of behavior. Based on the above analysis, there are the following hypotheses:

Hypothesis 1 (H1a): Standardized farmer professional cooperative affects the safety production behavior.
Hypothesis 1a: The restraint factors of farmer professional cooperatives have a positive impact on safety negative behavior.
Hypothesis 1b (H1b): The restraint factors of farmer professional cooperatives have a negative impact on safety positive behavior.
Hypothesis 1c (H1c): The motivation factors of farmer professional cooperatives have a negative impact on safety negative behaviors.
Hypothesis 1d (H1d): The motivation factors of farmer professional cooperatives have a positive impact on safety positive behavior.

#### Standardized farmer professional cooperative and safety motivation

Christiaans (38) and Bramall (39) found that the management factors of standardized farmer professional cooperatives have a more direct impact on farmers' safety motivation than other factors. Standardized farmer professional cooperatives have standardized management systems and can reduce farmers' unsafe production behaviors. Li and Ma (40) proposed that standardized farmer professional cooperatives have a dual stimulation effect on farmers' safety motivation. Farmers will not only be constrained by relevant regulations of standardized farmer professional cooperatives, but also get support in terms of capital, technology, and knowledge due to their participation in the organization. Giagnocavo et al. (41) pointed out that the premise of promoting agricultural supply-side structural reform is to improve the safety level of agricultural products from the source, and the source control of the quality and safety of various agricultural production subjects is the basis of realizing agricultural reform. Under the existing rural land system, farmer specialized cooperatives are an important carrier for Chinese small farmers to cope with the challenges of modern market. Farmer specialized cooperatives have a positive impact on farmers' safety motivation. According to the Triadic Reciprocal Determinism theory, the management factors of standardized farmer professional cooperatives belong to environmental factors, while the safety motivation belongs to the psychological factors of farmers. Individual psychological factors will be affected by environmental factors, individual perception has the role of stimulating and maintaining behavior, behavior will be affected by individual cognitive level. Based on the above analysis, there are the following hypotheses:

Hypothesis 2: Standardized farmer professional cooperative has an impact on safety motivation.
Hypothesis 2a (H2a): The restraint factors of farmer professional cooperative have positive impact on safety controlled motivation.
Hypothesis 2b (H2b): The restraint factors of farmer professional cooperative have negative impact on safety autonomous motivation.
Hypothesis 2c (H2c): The motivation factors of farmer professional cooperative have negative impact on safety controlled motivation.
Hypothesis 2d (H2d): The motivation factors of farmer professional cooperative have positive impact on safety autonomous motivation.

#### Safety motivation and safety production behavior

Safety motivation refers to people's willingness to perform work in a safe way, which is manifested as the motivation for safe production behavior (42). Human motivation can be divided into two types: control motivation and autonomous motivation. Different types of safety motivation may have different effects on human work safety behavior. Safety intervention from the motivation path is an effective management way. Mahdi et al. (43) believe that safety motivation is an important determinant of maintaining workplace safety behavior, autonomous safety motivation can significantly positively predict work safety behavior, and controlled safety motivation has significantly negatively predicted work safety behavior. Sok et al. (44) hold that safety motivation is a catalyst for farmers to voluntarily inoculate. Baur et al. (45) believe that safety motivation is the key factor for farmers to adhere to the practice of sustainable production, and the impact of safety motivation on the safety production behavior of farmers. According to the Triadic Reciprocal Determinism theory, Individual safety motivation and safety production behavior interact with each other. Individual perception can stimulate and maintain behavior, and behavior will be affected by individual cognitive level. Due to the difference in individual thinking and cognition, individuals will have different behaviors. The feedback effect of behavior in turn will reconstruct individual thinking and cognition. Based on the above analysis, there are the following hypotheses:

Hypothesis 3: Safety motivation has an effect on safety production behavior.
Hypothesis 3a (H3a): Safety controlled motivation has a positive effect on safety negative behavior.
Hypothesis 3b (H3b): Safety controlled motivation has a negative effect on safety positive behavior.
Hypothesis 3c (H3c): Safety autonomous motivation has a negative effect on safety negative behavior.
Hypothesis 3d (H3d): Safety autonomous motivation has a positive effect on safety positive behavior.

#### The intermediary role of safety motivation

According to the three-dimensional interaction theory, the environment affects individual psychological characteristics and individual psychological characteristics affect human behavior (46). In the environment of farmers' professional cooperatives, the psychological characteristics of farmers are affected by their motivition factors and restraint factors, which then produce safety motivation and ultimately affect the safety production behavior. The farmer professional cooperative is changing from the loose to the standard type, and gradually forms the standard organization with the nature of the enterprise. The technical training, the agricultural system supply, the brand strategy, and the recovery test of the farmer professional cooperative has an important influence on the farmer's production behavior (1, 47–50). Farmer professional cooperative is an important carrier to deal with the challenge of the modern market under the existing rural land system, and the farmer professional cooperative plays an important role in the process of farmers' production (51). According to the Triadic Reciprocal Determinism theory and Behavior-motivation theory, the management factors of standardized farmer professional cooperatives belong to environmental factors, the safety motivation belongs to the psychological factors of farmers, and the safety production behavior belongs to the behaviors taken by farmers. The management factors of standardized farmer professional cooperatives affect the safety motivation, and then affect the behavior of farmers. Safety motivation is the intermediary variable between management factors of standardized farmer professional cooperatives and the safety production behavior of farmers. Based on the above analysis, there are the following hypotheses:

Hypothesis 4: Safety motivation plays an intermediary role between farmer professional cooperatives and safety production behavior.
Hypothesis 4a (H4a): Safety controlled motivation plays an intermediary role between the restraint factors and the safety negative behaviors.
Hypothesis 4b (H4b): Safety controlled motivation plays an intermediary role between the restraint factors and the safety positive behaviors.
Hypothesis 4c (H4c): Safety autonomous motivation plays an intermediary role between the motivation factors and the safety negative behaviors.
Hypothesis 4d (H4d): Safety autonomous motivation plays an intermediary role between the motivation factors and the safety positive behaviors.

**Figure 1:**
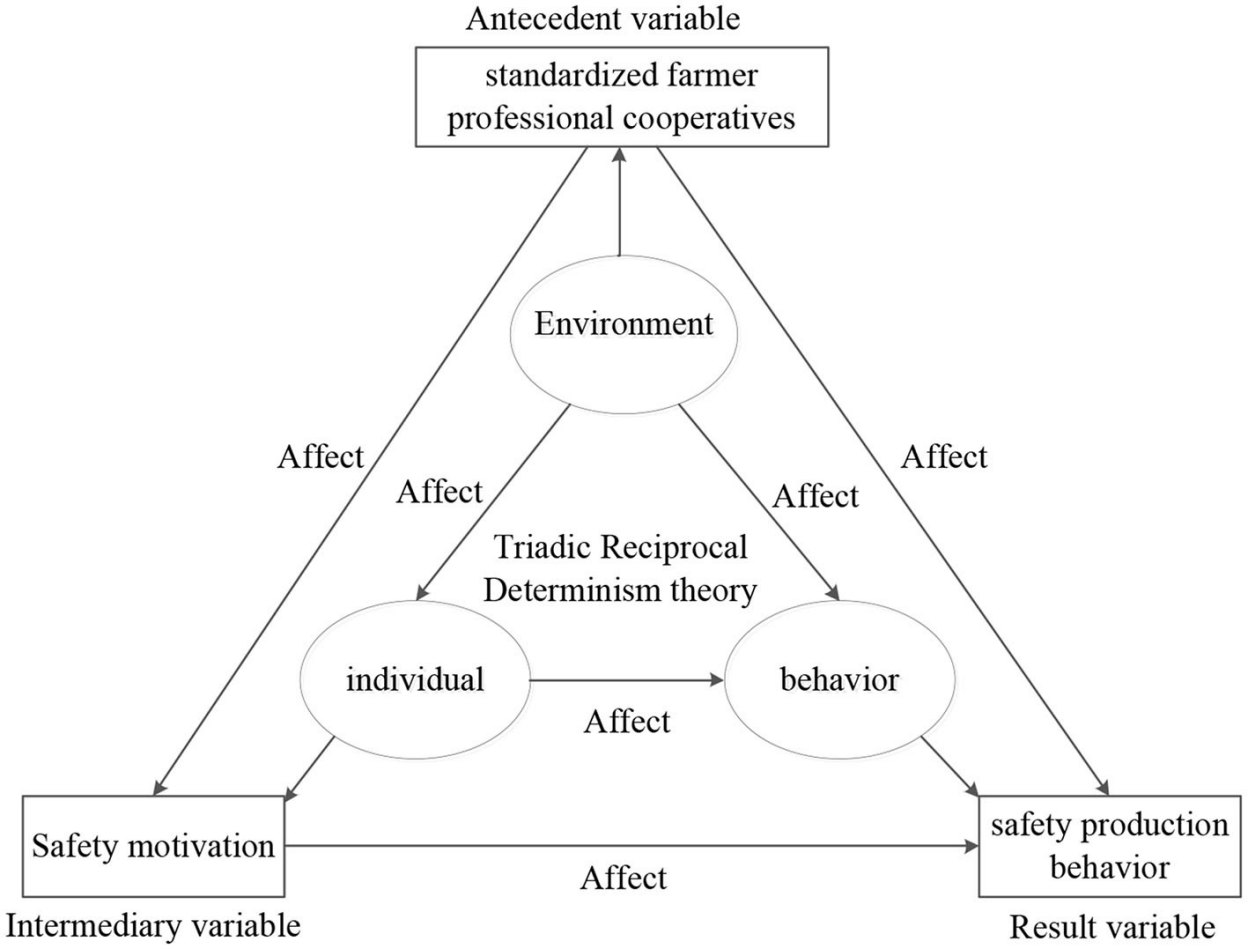
The functional relationship among standardized farmer professional cooperatives, safety motivation and safety production behavior.

According to the Triadic Reciprocal Determinism theory, the functional relationship among standardized farmer professional cooperatives, safety motivation, and safety production behavior can be fully explained, as shown in Figure 1.

**Figure 2:**
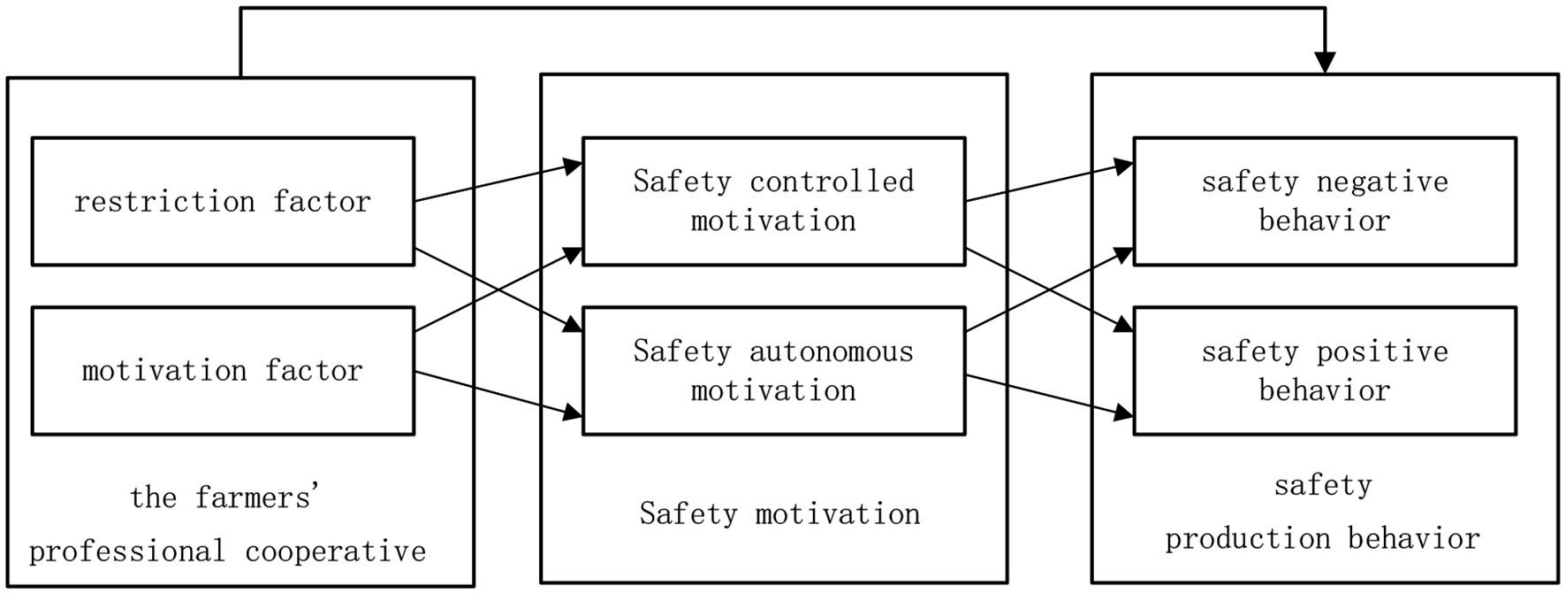
Hypothesized model.

Based on the above research hypothesis, the theoretical conceptual model of this study is shown in Figure 2.

## Research methodology

### Population and sample

This study follows the principle of region and classification, along the path of “East, middle, western regions, typical agricultural provinces, counties (districts, cities), farmer professional cooperative and social members,” and combines stratified sampling and household survey. A total of 800 members of 12 farmer professional cooperatives in China with family rules were investigated, covering five cooperatives of planting, breeding, forestry, animal husbandry, and aquaculture. Before the formal investigation, 2 standardized farmer professional cooperatives' members (*N* = 80) were selected to carry out the survey. We adopted a combination of stratified random sampling and random sampling to conduct large-scale questionnaire surveys with vegetable farmers in Heilongjiang Province, Jilin Province, and Liaoning Province. During the survey, the administrative areas of prefecture-level cities under the jurisdiction of each province are selected as the sampling cities (districts). Two townships are randomly selected from each city (District), one administrative village is randomly selected from each township, and several qualified farmers are randomly selected from each village. The questionnaire was revised by the sample data. The questionnaire was revised and a formal investigation was carried out. The time for formal field research is from October 2017 to December. The data collection process was supported and cooperated by the cooperative, and all questionnaires were sent out on the same day and collected on the same day. There were 800 questionnaires collected with a recovery rate of 100%. Through the study of 800 questionnaires, 767 valid questionnaires were obtained, with an effective rate of 95.9%. The basic information of the respondents is shown in Table 1 in the annex.

**Table 1:** Demographics of the sample (*n* = 660).

**Characteristic**	**% of sample**	**Characteristic**	**% of sample**	**Characteristic**	**% of sample**	**Characteristic**	**% of sample**
**Gender**		**Age**		**Planting years**		**Educational Level**	
Female	60.8	20–30 years	0.7	<10 years	20.6	Less than primary school education	38.2
Male	39.2	31–40 years	13.7	10–20 years	30.5	primary school education	44.1
		41–50 years	14.2	21–30 years	29.2	Secondary school education	16.6
		51–60 years	34.6	31–40 years	19.7	University and above education	1.0
		Over 60 years	36.8	Over 40 years			

### Survey instrument

The scale of this study is as follows, as shown in the Appendix:

1. Based on the research results of some researches, the revised scale contains 14 items (8 restraint factors, 6 motivation factors) combined with China's national conditions and research topics;
2. The safety production behavior refers to the scale of Griffin and Hoffman, combined with the national conditions and research topics of China, and the revised scale contains 10 items (5 safety controlled motivation, 5 safety autonomous motivation). With reference to the research results, and combined with the national conditions and research topics of China, the revised scale contains 8 items (4 safety negative behaviors and 4 safety positive behaviors). All scales were measured by Likert 1-5 scale, from 1 (totally disagree) to 5 (totally agree).

### Data analysis

In this study, SPSS and AMOS24.0 statistical analysis software were used to analyze the collected data, which mainly included a reliability test, validity test, and research hypothesis test.

## Research results

### Control and test of common method bias

The anonymous answer method and item meaning concealment method are used to control common method bias. The Harmanda factor test showed that the first principal component was 23.11% when it was not rotated, and the problem of homology is small and negligible.

### KMO test

SPSS 25.0 software was used to test the reliability and validity of the questionnaire, and the specific data are shown in Table 2. Reliability testing uses Cronbach's alpha as an indicator, and Cronbach's alpha values of all variables are >0.8, indicating that the scale has high internal consistency and good reliability. The values of the factor loading meet the requirements of being between 0.5 and 0.95, and there is a significant difference at the level of *P* < 0.01. In this study, exploratory factor analysis was used to study the structural validity of the initial scale of the questionnaire, and KMO test and Bartlett sphericity test were used to analyze the test results. The test results are shown in Table 2. The KMO measurement value is 0.890 (> 0.7), which meets the premise requirements of factor analysis. The approximate chi square of Bartley's spherical test is 10287.764, the degree of freedom is 666, and the value *P* is 0.000, <0.01. It has passed the significance test with a significance level of 1%. Therefore, it can be seen that the data is very suitable for factor analysis.

**Table 2:** Exploratory factor analysis KMO and Bartlett test.

**KMO**		**0.890**
Bartlett sphericity test	Approximate chi square	10287.764
	degree of freedom	666
	*P*	0.000

### Test of reliability and validity

Cronbachs' a coefficient was mainly used for the reliability test. Cronbachs' a coefficient of all scales in Table 3 was >0.7, indicating good reliability of the scale. The validity of the scale was tested from the content validity, convergence validity, and discriminant validity. The T value has reached a significant level, which shows that all the constructs have higher convergence validity. The correlation coefficient method is used to determine the validity of the discriminant validity. The 95% confidence interval of the correlation coefficient between the construct does not contain 1, and all the indexes of discriminant validity reach the acceptable level.

**Table 3:** The reliability and validity.

	**Construct**	**Reliability**	**AVE**	**Fitting index**
farmer professional cooperative (FPC)		0.927		
	Motivation factor (MF)	0.854	0.74	*NC* = 2.66 *RMSEA*(0.075) *NNFI*(0.94) *CFI*(0.93) *GFI*(0.95)
	Restraint factor (RF)	0.869		
Safety motivation (SM)		0.910		
	Safety controlled motivation (SCM)	0.858	0.71	*NC*=2.27 *RMSEA*(0.073 *NNFI*(0.96) *CFI*(0.92) *GFI*(0.94)
	Safety autonomous motivation (SAM)	0.837		
Safety production behavior (SPB)		0.932		
	Safety negative behavior (SNB)	0.783	0.77	*NC*=2.49 *RMSEA*(0.073) *NNFI*(0.92) *CFI*(0.96) *GFI*(0.94)
	Safety positive behavior (SAB)	0.841		

### Descriptive statistical analysis

The demographic variable is set as a virtual variable, which is processed into the contrast assignment between the different levels of the classified variables. The correlation coefficient of the demographic variables and the main variables is not extended. The main variables in the hypothesis are all relevant, as shown in Table 4.

**Table 4:** The descriptive and correlation analysis results.

	**Sex**	**Age**	**Entry age limit**	**Educational level**	**MF**	**RF**	**SCM**	**SAM**	**SNB**	**SAB**
Sex	/									
Age	0.211	/								
Entry age limit	0.118^*^	0.314^**^	/							
Educational level	0.218	−0.207^*^	0.252^**^	/						
MF	−0.104	0.201	0.156	0.214	(0.871)					
RF	0.208	0.314^*^	0.231^*^	0.228	−0.744^**^	(0.882)				
SCM	−0.114	0.032	0.091^*^	−0.209^*^	−0.619^**^	0.599^**^	(0.817)			
SAM	0.235	0.169^*^	0.197	0.188^**^	0.689^**^	−0.628^**^	−0.713^**^	(0.842)		
SNB	−0.130^*^	0.015	0.200^*^	0.222^*^	−0.571^**^	0.639^**^	0.699^**^	−0.636^**^	(0.833)	
SAB	0.095	0.183^**^	0.177	−0.389^**^	0.566^**^	−0.637^**^	−0.451^**^	0.711^**^	−0.774^**^	(0.823)

* P < 0.05.

** P < 0.01.

***P < 0.001.

### Hypothesis effect test

The effect test on the main variable is shown in Table 5.

**Figure 3:**
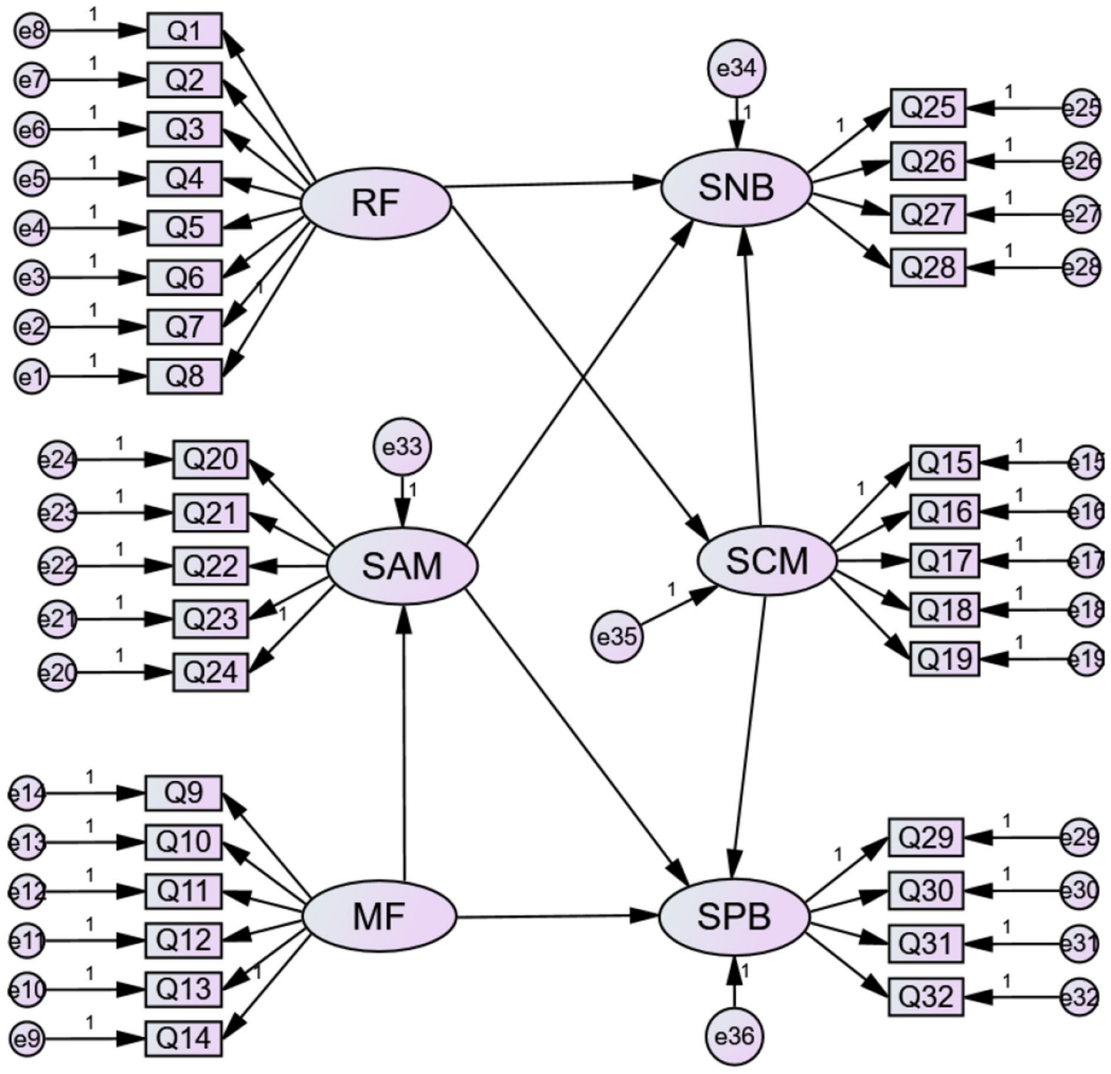
Mediation effect diagram.

1. Farmer professional cooperative does regression analysis on safety production behavior. In hypothesis 1a and hypothesis 1b, the regression coefficients of the restraint factors for the farmer professional cooperatives are, respectively (β = 0.682, *P* < 0.01) and (β = −0.529, *P* < 0.01). The interpretation effect *R*^2^ are 0.319 and 0.421, respectively. In the hypothesis 1c and hypothesis 1d, the regression coefficients of the motivation factors for the farmer professional cooperatives are, respectively (β =0.277, *P* > 0.05) and (β =0.739, *P* < 0.01), and the interpretation effect *R*^2^ are 0.323 and 0.318, respectively. Hypothesis 1a, 1b, and 1d are established, and hypothesis 1c is not valid.
2. Regression analysis of farmer professional cooperative on safety controlled motivation and safety autonomous motivation. In hypothesis 2a and hypothesis 2b, the regression coefficients of the restraint factors of farmer professional cooperatives to safety negative behavior and safety positive behavior are (β = 0.680, *P* < 0.01), (β = −0.414, *P* < 0.01), and the interpretation effect *R*^2^ are 0.465 and 0.384, respectively. In the hypothesis 2c and hypothesis 2d, the regression coefficients of the motivation factors for the farmer professional cooperatives are, respectively (β = 0.107, *P* > 0.05) and (β = 0.729, *P* < 0.01), and the interpretation effect *R*^2^ are 0.299 and 0.337, respectively. Hypothesis 2a, 2b, and 2d are established, and hypothesis 2c is not valid.
3. Safety motivation is a regression analysis of safety production behavior. In hypothesis 3a and hypothesis 3b, the regression coefficients of safety controlled motivation to safety negative behavior and safety positive behavior are (β = 0.605, *P* < 0.01) (β = −0.148, *P* > 0.05), and the interpretation effect *R*^2^ are 0.424 and 0.386, respectively. In hypothesis 3c and hypothesis 3d, the regression coefficients of safety autonomous motivation to safety negative behavior and safety positive behavior are (β = −0.493, *P* < 0.01) (β = 0.774, *P* < 0.01), and the interpretation effect *R*^2^ are 0.463 and 0.401, respectively. Hypothesis 3a, 3c, and 3d are established, and hypothesis 3b is not valid.
4. Mediation model validation. The Sobel test is a commonly used test method in mediating model validation, but research shows that the Sobel test has certain limitations. The bootstrap technique is a method that repeatedly samples from samples, and it has a more accurate confidence interval and a higher test process than the Sobel method. Thus, we adopt the bootstrap method to test the mediating effects proposed in hypothesis 4. The results are presented in Figure 3 and Table 6. The regression coefficients of the safety controlled motivation to the safety negative behavior and the safety positive behavior are, respectively (β = 0.402, *P* < 0.01; β = 0.402, *P* > 0.05). The regression coefficients of safety autonomous motivation to safety negative behavior and safety positive behavior are (β = −0.171, *P* > 0.05; β = 0.405, *P* < 0.01). Only the safety controlled motivation reduces the regression coefficient of the safety passive behavior and the safety autonomous motivation to the safety positive behavior, which indicates that the safety controlled motivation plays a partial intermediary role between the farmer professional cooperative and the safety negative behavior, and the safety autonomous motivation plays a partial intermediary role between the farmer professional cooperative and the safety positive behavior. Hypothesis 4a and hypothesis 4d are established, and hypothesis 4b and hypothesis 4c are not valid.

**Table 5:** Hypothesis test result.

**Hypothesis**		**Relationship**	**β**	***P***	***R* ^2^**	**F**	**Positive or negative correlation**
Hypothesis 1	H1a	RF→ SNB	0.682	<0.01	0.319	34.372^**^	Positive correlation
	H1b	RF→ SPB	−0.529	<0.01	0.421	30.626^**^	Positive correlation
	H1c	SF→ SNB	0.277	>0.05	0.323	35.219^**^	Negative correlation
	H1d	SF→ SPB	0.739	<0.01	0.318	38.984^**^	Positive correlation
Hypothesis 2	H2a	RF→ SCM	0.680	<0.01	0.465	34.464^**^	Positive correlation
	H2b	RF→ SAM	−0.414	<0.01	0.384	30.328^**^	Positive correlation
	H2c	SF→ SCM	0.107	>0.05	0.299	32.581^**^	Negative correlation
	H2d	SF→ SAM	0.729	<0.01	0.337	28.904^**^	Positive correlation
Hypothesis 3	H3a	SCM→ SNB	0.605	<0.001	0.424	30.101^**^	Positive correlation
	H3b	SCM→ SPB	−0.148	>0.05	0.386	37.099^**^	Negative correlation
	H3c	SAM→ SNB	−0.493	<0.001	0.463	30.884^**^	Positive correlation
	H3d	SAM→ SPB	0.774	<0.001	0.401	36.804^**^	Positive correlation

** *P* < 0.01.

**Table 6:** Results of the mediation test.

**Hypothesis**		**Relationship**	**β**	**SE**	**P**	**95% CI**
Hypothesis 4	*H4a*	RF→ SCM→ SNB	0.402	0.023	<0.01	[0.02, 0.36]
	*H4b*	RF→ SCM→ SPB	0.402	0.048	> 0.05	[−0.30, −0.11]
	*H4c*	MF→ SAM→ SNB	−0.171	0.051	> 0.05	[−0.39, −0.17]
	*H4d*	MF→ SAM→ SPB	0.405		<0.01	

## Discussion

Based on the Triadic Reciprocal Determinism theory, this study verifies the impact of standardized farmer professional cooperatives on safe production behavior. The results can be summed up as follows:

(1) The standardized farmer professional cooperative has an impact on the safety production behavior, in which the restraint factors positively affect the safety negative behavior, the restraint factors negatively affect the safety positive behavior, and the motivation factors positively affect the safety positive behavior. (2) The farmer professional cooperative has an impact on the safety motivation, in which the restraint factors are positively affecting the safety controlled motivation, the restraint factors of the farmer professional cooperative are negatively affecting the autonomous motivation, and the motivation factors of the farmer professional cooperative are positively affecting the safety autonomous motivation. (3) Safety motivation has an impact on the safety production behavior, in which the safety controlled motivation positively affects the safety negative behavior, the safety autonomous motivation negatively affects the safety negative behavior, and the safety autonomous motivation is positively affecting the safety positive behavior. (4) The safety motivation plays a mediating role between the farmer professional cooperative and the safety production behavior, in which the safety controlled motivation plays a mediating role between the restraint factors of the farmer's professional cooperative and the safety negative behaviors, and the safety autonomous motivation plays a mediating role between the motivation factors of the farmer's professional cooperative and the safety negative behavior.

Research results showed that the farmer professional cooperative motivation factors on safety negative behavior and controlled motivation will not produce negative effects (Hypothesis 1c, 2c, and 3b). The motivation factors can more mobilize the enthusiasm of farmers to perform safety behavior, and then promote farmers to take safe behavior, motivation factors can more mobilize the enthusiasm of farmers than restraint factors, and promote farmers to take safe behavior. Safety controlled motivation cannot play an intermediary role between the restraint factors and safety positive behaviors (Hypothesis 4b). Safety autonomous motivation cannot play an intermediary role between the motivation factors and the safety negative behaviors (Hypothesis 4c).

The main innovation points of this study are as follows: The existing research methods for farmers' production behavior are mostly limited to traditional methods such as questionnaires and interviews, and the traditional survey and interview methods cannot clearly explore the complex game situation between actors. In addition, existing studies mostly focus on the impact of government and enterprises, cooperatives, and consumers on the quality and safety of agricultural products, and lack of studies related to cooperatives. In the later period, research methods gradually increase, but there are still few studies related to cooperatives. As rational economic people, farmers' production behaviors will be influenced by whether their green production behaviors can bring greater economic benefits, whether the government supervises their production behaviors, and whether cooperatives standardize their behaviors. The purpose of the research is to improve the quality and safety of agricultural products from the source, and study closely the realistic problems. Farmers' production behavior based on the quality and safety of agricultural products is a theoretical and practical problem with great practical significance. Different from the previous many studies, the Research Topic is focused on determining the source of the quality and safety of agricultural products, rising from the static characteristics of the farmers' production behavior to the dynamic characteristics. The “making farmers produce quality and safety agricultural products” is extended to “farmers to produce quality and safety agricultural products.” From the perspective of standardized farmer professional cooperative, this study introduces the variable of safety motivation, divides the regulatory factors of farmer professional cooperative into restraint factors and motivation factors, divides the safety motivation into safety controlled motivation and safety autonomous motivation, and divides safety production behavior into safety negative behavior and safety positive behavior. Empirical studies verify the relationship between these complex variables.

The content of the study focuses on the support theory and the hypothesis model. Based on the Triadic Reciprocal Determinism theory and Behavior-motivation theory of the social cognition school, this study concludes that the regulatory factors of the standardized farmer professional cooperative belong to the environmental factors. The transition of the safety production behavior belongs to the behavioral factors, and the safety motivation belongs to the psychological factors, based on the typical theory of “environmental impact psychological, psychological impact behavior” and “motivational behavior” Based on this, the research hypothesis model is put forward, and the support theory and the research hypothesis model are verified. This study explores the mechanism of the influence of the management factors of farmer professional cooperative on the safety production behavior, and provides a practical basis for the construction of the management mechanism of farmer professional cooperative, ensuring that the mechanism meets the practical needs, and promotes the farmers' positive production of quality and safety of agricultural products.

## Conclusion

The quality and safety of agricultural products is an important breakthrough in the upgrading of agricultural transformation in the world. The key to realizing the quality and safety of agricultural products is the control of the source of production. The rapid development of farmer professional cooperatives has become an effective way to improve the quality and safety of agricultural products. farmer professional cooperative promotes the systematization of farmers' groups, reconfigures production decisions, and achieves economies of scale development. Under the mode of institutionalization and organization, the restraints and motivations of farmer professional cooperatives can effectively control and improve the quality and safety of agricultural products. Through the study, it is found that the restraint factors and motivation factors of farmer professional cooperatives can affect safety negative behavior and safety positive behavior through safety controlled motivation and safety autonomous motivation.

First, farmer professional cooperatives should formulate reasonable restraint factors and motivation factors. With the increasing demand for agricultural products, agricultural products are facing the current situation of short supply. In order to improve the output, the farmers pursue economic benefits, misuse or excessive use of pesticides and chemical fertilizers, and the quality and safety of agricultural products cannot be guaranteed in the source. The management factors of farmer professional cooperatives can effectively control farmers' production behavior, and the management factors of farmer professional cooperatives mainly involve factors such as technical training, purchase agreement, agricultural system supply, unified control, brand strategy, recovery testing, and the formulation of protective prices. The rational combination of these factors can mobilize the motivation of farmers' safety and autonomy, reduce the motivation of safety and autonomy, and thus increase the safety positive behavior and reduce the safety negative behavior. farmer professional cooperative needs to further broaden the field of cooperation, seek cross-regional and cross-industry cooperation, and form the quality and safety of agricultural products industry chain. At the same time, the farmer professional cooperative must establish a perfect quality and safety inspection system for agricultural products, standardize the inspection standards for agricultural products, establish a scientific system testing process, provide professional inspectors, and strictly control the quality and safety of agricultural products. The farmer professional cooperative considers establishing a traceable system for the quality and safety of agricultural products, which can be quickly traced to the producers for the detection of unqualified agricultural products in order to take appropriate measures.

Second, the establishment of a pluralistic governance mechanism is the only way to promote farmers' initiative in producing quality and safety agricultural products. The transformation from “managing the country” to “governing the country” and from “management thought” to “governance thought” has provided useful inspiration for solving farmers' production problems internationally. At present, the economic and social development of many countries is in a complex environment of multi-center interdependence. Agricultural production is dominated by large quantity and small-scale scattered farmers. The government cannot supervise and control the farmers' safety production behavior in all directions. Besides the farmer professional cooperatives, it is necessary to give full play to the complementary advantages of the government, the market, and the society, to build a multi-governance mechanism for the safety production behavior, and to promote the farmers' passive transfer to the positive production of quality and safety agricultural products. This is the support from the theory and the reflection from the practice.

Third, farmer professional cooperatives may regularly contact agricultural scientific research institutions, organize professional personnel to provide practical guidance on green production technologies for farmers, and popularize innovative technologies in rural areas; Cooperatives appropriately increase dividends to farmers of green production and increase the punishment for farmers of non-green production, so as to encourage farmers to take the initiative in green production. Farmer professional cooperatives should unify production standards and agricultural supplies, and supplement with human capital cultivation and organizational culture construction, so as to make management methods scientific, standardized and systematic, and build a modern management mechanism of self-restraint, self-perfection, and self-motivation.

Fourth, farmers should be aware of the importance of their own behavior and the impact of their produce on consumers' physical and mental health, and actively respond to the government's policies and calls. As for the rules and regulations formulated by farmer professional cooperatives, farmers should actively cooperate to carry out green production according to standards, provide high-quality agricultural products, and actively participate in the technical training organized by cooperatives to improve their awareness of green production, reduce their dependence on traditional pesticides, and fundamentally solve the quality and safety problems of agricultural products.

The theoretical contribution of this study is to put forward the green production behavior of farmers based on the quality and safety of agricultural products and divide farmers' production behavior into green and non-green production behavior, which broadens the research perspective. Combined with social cognition theory and behavioral motivation theory, it is proposed that farmers' behavior should be divided into two dimensions, non-green production behavior and green production behavior, which expands the relevant theory of agricultural product quality and safety. The limitation of this paper is the selection of samples, and there are certain limitations in the type and breadth of cooperatives selected, so the investigation scope of cooperatives should be expanded in future research.

## Data availability statement

The original contributions presented in the study are included in the article/supplementary material, further inquiries can be directed to the corresponding author.

## Ethics statement

The studies involving human participants were reviewed and approved by Northeast Agricultural University. The patients/participants provided their written informed consent to participate in this study.

## Author contributions

YT contributed to the conception and design of the study and wrote the first draft of the manuscript. XC organized the database. MZ performed the statistical analysis. XC and MZ wrote sections of the manuscript. All authors contributed to manuscript revision, read, and approved the submitted version.

## Funding

This work was supported by Humanities and Social Sciences Foundation of Ministry of Education of China (Grant 18YJC630162), Heilongjiang and Province Postdoctoral Science Foundation (Grant LBH-Z107018), Key Laboratory Project of Modern Agricultural Equipment Technology in Northern Cold Region (Grant KF18-01), Young Talents of Northeast Agricultural University (Grant 20XG07), and Heilongjiang Philosophy and Social Sciences Research Planning Project (Grant 21JYD273), and Heilongjiang Social Science Fund Project (Grant 21JYB150).

## Conflict of interest

The authors declare that the research was conducted in the absence of any commercial or financial relationships that could be construed as a potential conflict of interest.

## Publisher's note

All claims expressed in this article are solely those of the authors and do not necessarily represent those of their affiliated organizations, or those of the publisher, the editors and the reviewers. Any product that may be evaluated in this article, or claim that may be made by its manufacturer, is not guaranteed or endorsed by the publisher.

## Appendix

**Table:** The construction of elements scale.

**Variable**	**Dimension**	**Measurement item**
Management factors of standardized farmer professional cooperative	Restraint factors	1. The supervision of standardized farmer professional cooperatives.
		2. Standardized farmer professional cooperatives manage their members.
		3. Standardized farmer professional cooperatives take punitive measures against their members.
		4. Standardized farmer professional cooperatives shall investigate the responsibility for the quality of agricultural products.
		5. Standardize the quality supervision of farmer professional cooperatives on seeds.
		6. Standardized farmer professional cooperatives regularly emphasize the use of chemical fertilizers and pesticides.
		7. Standardized farmer professional cooperatives require farmers to record the varieties of chemical fertilizers and pesticides and relevant information.
		8. Regular technical training for members of standardized farmer professional cooperatives.
	Motivation factors	1. Standardized farmer professional cooperatives shall take incentive measures to reward members.
		2. Members of standardized farmer professional cooperatives receive dividends
		3. Standardized farmer professional cooperatives give members certain subsidies
		4. Standardized farmer professional cooperatives ensure the safety of planting bases.
		5. Standardized farmer professional cooperatives buy back agricultural products with quality problems.
		6. Standardized farmer professional cooperatives purchase seeds or chemical fertilizers for their members.
Safety production behavior	Safety controlled motivations	1. Your family's economic status will affect your safety production behavior.
		2. Your neighbors or friends take safety production actions.
		3. Government and legal control will affect your safety production behavior.
		4. The development of social environment will affect your safety production behavior.
		5. The profit incentive of the market will affect your safety production behavior.
	Safety autonomous motivations	1. You will take the initiative to take safe production actions.
		2. You will advise your members to take safe production behavior
		3. You will take the initiative to care about how to take safe production behavior.
		4. Your education level supports you to adopt safe production behavior.
		5. You know how to take safe production behavior.
Security motivation	Safety negative behaviors	1. You will not actively learn new safety knowledge.
		2. You will not actively participate in education and training.
		3. You will not actively participate in security affairs.
		4. You will not actively participate in the establishment or change of new rules and regulations.
	Safety positive behaviors	1. You will actively learn new safety knowledge.
		2. You will actively participate in education and training.
		3. You will actively participate in security affairs.
		4. You will actively participate in the establishment or change of new rules and regulations.
